# Insect Gallers and Their Plant Hosts: From Omics Data to Systems Biology

**DOI:** 10.3390/ijms17111891

**Published:** 2016-11-18

**Authors:** Caryn N. Oates, Katherine J. Denby, Alexander A. Myburg, Bernard Slippers, Sanushka Naidoo

**Affiliations:** 1 Department of Genetics, Forestry and Agricultural Biotechnology Institute (FABI), Genomics Research Institute (GRI), University of Pretoria, Private Bag x20, Pretoria 0028, South Africa; caryn.oates@fabi.up.ac.za (C.N.O.); Zander.Myburg@up.ac.za (A.A.M.); Bernard.Slippers@up.ac.za (B.S.); 2 Department of Biology, University of York, Wentworth Way, York YO10 5DD, UK; katherine.denby@york.ac.uk

**Keywords:** galling insect, omics data, effector, phytohormone, gall induction

## Abstract

Gall-inducing insects are capable of exerting a high level of control over their hosts’ cellular machinery to the extent that the plant’s development, metabolism, chemistry, and physiology are all altered in favour of the insect. Many gallers are devastating pests in global agriculture and the limited understanding of their relationship with their hosts prevents the development of robust management strategies. Omics technologies are proving to be important tools in elucidating the mechanisms involved in the interaction as they facilitate analysis of plant hosts and insect effectors for which little or no prior knowledge exists. In this review, we examine the mechanisms behind insect gall development using evidence from omics-level approaches. The secretion of effector proteins and induced phytohormonal imbalances are highlighted as likely mechanisms involved in gall development. However, understanding how these components function within the system is far from complete and a number of questions need to be answered before this information can be used in the development of strategies to engineer or breed plants with enhanced resistance.

## 1. Introduction

The ability to induce galls is a specialised feeding behaviour that requires a close host-pest adaptation [1]. Insect galls are abnormal plant growths that are induced and maintained by the pest and are characterised by active redifferentiation and growth of plant tissues [1]. The insect is capable of redirecting normal plant metabolism and physiology towards gall production [2,3,4]. How the insect is able to achieve such an extraordinary level of control over its host is perhaps the most intriguing question surrounding plant-galling insect interactions and has yet to be conclusively demonstrated for any species. 

Gall-inducing arthropods and nematodes comprise some of the most devastating pests of global agriculture. These include interactions between phylloxera (*Daktulosphaira vitifolia*) and grape (*Vitis vinifera*), the Hessian fly (*Mayetiola destructor*) and wheat (*Tritcum aestivum*), the Asian rice gall midge (*Orseolia oryzae*) and rice (*Oryza sativa*), the blue gum chalcid wasp (*Leptocybe invasa*) and *Eucalyptus* spp., as well as root-knot nematodes (*Meloidogyne* spp.) and a wide range of hosts amongst numerous other examples. The development of resistant plant genotypes through breeding or genetic modification is a commonly used approach to control these pests; however, resistance is often rapidly overcome by more virulent insect biotypes [5]. The lack of information regarding the molecular mechanisms behind plant-galling insect interactions is an impediment to the development of novel, robust management strategies.

In recent years, omics approaches have been applied to various host-pest interactions [6]. Next-generation omics approaches facilitate the analysis of non-model organisms owing to the rapid generation of large amounts of de novo systems biology data, making them attractive options for studying poorly-characterised interactions [7]. In this review, we discuss the current understanding of morphological and molecular mechanisms underlying gall formation and host defences based on evidence from omics data. We examine the concept of systems biology tailored to plant-galling insect interactions. Lastly we identify specific questions, to help elucidate the complex interaction, using a systems approach.

## 2. The Galling Trait

The ability to induce galls is an ancient life-history trait with fossil records supporting the ecological expansion of foliar galling during the Early Permian era, around 299–252 million years ago [8]. It has evolved multiple times in insect lineages [9] and an estimated 13,000 extant species are known to induce galls [1]. How this characteristic arose, particularly in the sense of the extensive ability to control their hosts’ cellular functioning, remains a subject of debate. One interesting hypothesis proposes that galling insects acquired genes through horizontal gene transfer from symbiotic microorganisms [10,11,12,13]. Many galling insects are known to have microbial associates that may be involved in gall development or facilitate herbivory, such as Ambrosia gall midges that are commonly associated with fungal symbionts [14,15]. Furthermore, many microbial symbionts are able to synthesise phytohormones, chemicals known to be key elements in the plant-galling insect interaction [11]. Since the trait evolved multiple times, it is likely that the evolutionary process differs amongst lineages. Regardless of how it arose, it is clearly a successful life-history trait.

### 2.1. Adaptive Significance of the Galling Trait

Enhanced nutrition is considered to be an important driving force behind the evolution of galling [1,16]. There is a large diversity amongst gall types, ranging from open pits or folds, to structures which completely encase the insect, but all contain a nutritive tissue that is formed by redifferentiation of plant tissues [1,16]. The composition and structure of this zone varies among species, but do present a number of general features [16]. The nutritive tissue is organelle-rich, frequently including visible nucleoli, enlarged or fragmented vacuoles, dense cytoplasm, and high numbers of Golgi apparatus, endoplasmic reticulum, and ribosomes, which are traits indicative of the high metabolic status of these cells [17,18,19,20]. Secondly, this tissue comprises high concentrations of lipids, proteins, and carbohydrates, which supports its function as an enhanced source of nutrition to the developing insect [21,22].

Gall-inducing insects are able to manipulate the source-sink dynamics within their hosts and reconfigure their host’s metabolism [14,23,24,25,26,27,28,29]. Transcriptomic studies of a variety of plant-galling insect interactions have described general up-regulated expression of primary metabolism and nutrient transport in the plant, as well as down-regulation of defence-associated processes. These studies include interactions between the blue gum chalcid and *Eucalyptus* [23], phylloxera and grape [25], the Asian rice gall midge and rice [26], and the Hessian fly and wheat [28]. 

The ability of gallers to actively modify source-sink relationships in their hosts is reflected in a number of metabolomics studies that demonstrate altered nutrient allocation patterns during galling. Nabity et al. [25] used carbon-14 labelling and mass spectrometry experiments to demonstrate sequestration of carbon-compounds from the surrounding grape leaf into the phylloxera-induced gall. Similar results were demonstrated by Compson et al. [27] in aphid (*Pemphigus betae*) galling of narrowleaf cottonwood (*Populus angustifolia*). Increased concentrations of sugars, carbon-containing compounds and nitrogen-containing compounds have also been reported for galling interactions between *Bruggmanniela* sp. (Diptera: Cecidomyiidae) and *Litsea acuminate* [14]. Together, these results appear to confirm the role of galls in providing enhanced nutrition to the insect. The expression profiles obtained from these studies support the role of galls as nutrient sinks and demonstrate the great level of control that the insect is able to exert on its host.

### 2.2. Development and Structure of Galls

Galls are formed by redifferentiation of host plant tissues and often develop through a combination of hypertrophy (increased cell size) and hyperplasia (increased cell numbers) which are processes mediated by plant growth regulators [30,31]. These processes are not well-understood in insect-induced galls, although inferences can be made from other species, such as root knot nematodes [32,33]. A number of histological studies have investigated insect gall development, which provides a foundation for designing and interpreting omics experimental data of these interactions. We, therefore, consider some important findings to emerge from these studies.

Oliveira and Isaias [3] provide a detailed description of the development of galls induced by an undescribed species of Cecidomyiidae (Diptera) on the diesel tree (*Copaifera langsdorffii*). The redifferentiation of specific plant tissues into the gall specific tissues is tracked over time and provides an example of the complexity of the interactions between the gall-inducer and its host plant. The primary plant cell wall is a complex structure formed by a network of cellulose microfibrils and hemicellulose embedded in a pectic polysaccharide matrix [34]. Insect-induced changes in the structure of the plant cell walls determine the final shape of the cell [30,35], but how the galling insect induces changes to host cell walls to produce a gall are poorly understood. 

Formiga et al. [34] investigated cell wall dynamics using three gall morphotypes (rolling, pocket, and kidney-shaped), induced by two unknown species of Psylloidea and one Cecidomyiidae, in *Baccharis reticularia*. The authors used monoclonal antibodies to detect pectins, glycoproteins, galactan, arabinan, and extensins, all important cell wall components, in each system. It was shown that pectin dynamics, which control cell wall flexibility and rigidity, play an important role in gall-tissue development and pectin modification enzymes may, therefore, be expected to show up in transcriptome studies of early gall development.

Suzuki et al. [30] investigated the subcellular localisation of a variety of cell growth regulators (reactive oxygen species, polyphenols and auxins) and cellulose microfibrils over the development of a gall induced by *Lopesia* spp. (Cecidomyiidae) on *Lonchocarpus cultratus*. These regulators occur at sites of cell hypertrophy. The simultaneous presence of reactive oxygen species and anti-oxidants indicate a chemical balance between the regulation of growth and the avoidance of cell death at gall sites [36]. Auxins have been frequently associated with galls; however, the molecular mechanisms that are involved are currently unknown.

## 3. The Molecular Mechanisms of Gall Induction

The molecular mechanisms of insect gall induction and maintenance are currently poorly understood. The unifying characteristic consistent across all galls and which separates galling from other modes of herbivory is the redifferentiation of host tissues into gall-specific tissues [1]. By extension, it is plausible that a fundamental requirement of galling requires the pest to assume control of the host’s cellular machinery and to maintain this control throughout the period of time that the insect is dependent on the host. Comparisons between different insect gallers and other galling species, such as root knot nematodes, have highlighted two mechanisms that putatively regulate this process: the secretion of effectors and induced phytohormonal imbalances.

### 3.1. Effectors in Insect Galling

An effector is a molecule with specific host targets that may allow the attacker to undermine the hosts’ immune system and modulate the cellular processes [37]. Jones and Dangl [38] proposed the currently accepted model of the resistance (R) protein-effector arms race between plants and pathogens. Plant pattern-triggered immunity (PTI) is initiated following the recognition of microbe-associated molecular patterns (MAMPs). PTI is suppressed by pathogen-secreted effectors (effector-triggered susceptibility, ETS). ETS is countered by the recognition of these effectors by intracellular R proteins (effector-triggered immunity, ETI). The arms race continues as the pathogen evolves new effectors to avoid detection by the plant and the plant evolves new R proteins to improve surveillance. This model is currently also accepted for plant-insect interactions [39] and herbivore- and egg-associated molecular patterns (HAMPs and EAMPs), as well as effectors that elicit PTI and ETI, respectively, have been identified [40,41,42,43,44]. Where plant genomes are available, putative *R* genes can be identified as clusters of polymorphic, nucleotide binding site leucine-rich repeats-containing genes [39]. The Hessian fly was the first insect proposed to have a gene-for-gene interaction with its host, a hypothesis that was confirmed upon the identification of the first galling insect effector [45].

Putative insect effector-encoding genes are commonly identified by four characteristics [40,46,47,48]; however, it must be noted that other parameters may also be considered in the identification of insect effectors [49]. Firstly, these genes show no sequence homology to known genes due to high selection pressure, leading to rapid sequence divergence; Secondly, they generally encode short (50–250 amino acids) oligopeptides. This size range should only be considered as a guideline as a number of effectors have been identified that are larger than 250 amino acids; Thirdly, putative insect effector-encoding genes frequently possess an N-terminal secretion signal; Finally, they exhibit localised expression in the earliest interfaces between the insect and the pest which is generally the saliva [50] and oviposition fluid [51]. Alternatively, putative insect effectors can be identified in the genome of the insects as clusters of highly variable genes [47].

A number of genomic and transcriptomic studies have focused on the identification of the effector repertoires of important plant parasites. Of these, the Hessian fly is the only galling species to have been examined at this multi-omics level. More than 7% of the Hessian fly genome is estimated to encode putative effector proteins, which includes the largest known arthropod gene family, secreted salivary gland protein (*SSGP*)*-71* [47]. Many of these putative effectors were first reported in transcriptomic studies and showed localised salivary gland expression in first instar larvae in earlier studies [52,53]. Furthermore, variation in the expression of *SSGPs* amongst field-collected larvae separated into groups that corresponded to the wheat classes grown in the different geographical regions, as well as recently described Hessian fly populations [54]. A second, integrative approach for identifying effector repertoires was shown in a comparative transcriptomics (head vs. body) and proteomics (saliva) study of three different aphid species [46]. The authors were able to identify putative effector sets that were unique to each species and shared amongst all three.

Another effector-based mechanism, whereby a gall-inducing insect may manipulate the transcriptional responses of the plant, is through the use of non-coding RNAs, such as microRNAs (miRNAs). These molecules play a critical role in regulating post-transcriptional expression [48]. Since miRNAs regulate gene expression by direct pairing, potential regulatory targets can also be predicted computationally by searching for complementary sequence similarity [48]. Deep sequencing of a Hessian fly larval transcriptome led to the identification of 89 known and 184 novel miRNA species [48]. An examination of a draft Hessian fly genome identified 611 putative miRNA-encoding genes based on sequence similarity and the existence of a stem-and-loop structure for miRNA precursors [48]. Microarray analyses on this set revealed a dramatic expansion of several miRNA gene families. Furthermore, expression of these miRNAs was strictly regulated during larval development and abundance of many miRNA genes was affected by host genotype [48]. 

The effects of a number of insect effectors, particularly in species of lepidopterans and aphids, on plant responses have been explored; however, the targets of these proteins and their molecular functions remain unknown [51]. One example of an integrative approach using omics data to identify putative effectors for functional testing was provided by Villarroel et al. [40] using species of non-galling spider mites. The authors performed in silico predictions of effector genes using the *Tetranychus urticae* (*T. urticae*) (generalist spider mite) genome and a de novo assembled transcriptome of *Tetranychus evansi* (*T. evansi*) (specialist spider mite), as well as life stage expression and salivary gland localisation as criteria. Four proteins from two families suppressed defences downstream of salicylic acid following transient expression in *Nicotiana benthamiana*.

With regards to the Hessian fly, Zhao et al. [47] showed that, although *SSGP-71s* lack sequence homology to other genes, their structures resemble plant ubiquitin E3 ligases and plant pathogenic E3 ligase-mimicking effectors. Some members contained F-box motifs that allowed them to interact with Skp-1 proteins, a component of the Skp-Cullin-F-box-E3-ubiquitin-RING-ligase complex that targets proteins for degradation. This provides a possible mechanism for the insect to hijack the plant proteasome and defeat basal immunity [47]; whether this is the case remains subject to future study.

Mukhtar et al. [55] described how known effectors from plant pathogens with diverse lifestyles (*Pseudomonas syringae* and *Hyaloperonospora arabidopsidis*) tend to target a relatively small number of hubs in the *Arabidopsis thaliana* (*A. thaliana*) immune system network. Hubs are highly interconnected points within a network where perturbations can have large effects on the downstream network. Hubs within the plant immune network that are targeted by pathogen or pest effectors may provide a means for manipulating a large proportion of the defence response, thereby enabling successful invasion. The robustness of the plant immune network is a counter-measure that allows the plant to launch a successful defence response in spite of a suit of effectors introduced by an invader [56]. Considering that galling insects must reprogram the host cellular machinery at both the developmental and immune levels (i.e., suppress plant defences whilst simultaneously generate gall-specific tissues), it seems logical to expect gallers to possess a wide array of effectors in order to achieve this.

### 3.2. Phytohormones in Insect Galling

Galls commonly grow via the mechanisms of hypertrophy or hyperplasia, or a combination thereof. Tooker and Helms [31] reviewed evidence regarding the role of various phytohormones in gall development and host manipulation, as well as plant resistance against galling insects. Two of the hormones addressed in the review, auxins and cytokinins, are well-known growth regulators that have been repeatedly implicated in insect gall development and their role in plant-galling insect interactions is a widely accepted hypothesis.

The value of obtaining omics data in systems that have not been previously characterised was recently demonstrated with a simple transcriptome comparison between galled and ungalled leaves of *Metrosideros polymorpha* (Myrtaceae) by *Trioza* spp. (Hemiptera: Triozidae), a galling psyllid [57]. The researchers de novo assembled transcriptomes of each treatment and were able to separate plant and insect contigs in silico, although only the plant transcriptome was analysed further. The most notable observation was the significant enrichment of auxin-response genes associated with galling in this system. No auxin synthesis genes were observed leading to the hypothesis that the hormone was introduced exogenously. A number of studies have proposed this same hypothesis; however, the lack of suitable labelling experiments and appropriate omics data means that distinguishing the source of the hormone has been difficult [31].

Metabolomic and transcriptomic approaches are commonly used to investigate changes in phytohormone concentrations, as well as hormone synthesis and responsive genes, during insect infestations. One example of this integrated approach was demonstrated by Zhang et al. [58]. The authors examined phytohormone dynamics during the interaction between apple (*Malus domestica*) and a leaf-mining lepidopteran, *Phyllonorycter blancardella* (Lepidoptera: Gracillariidae) using liquid chromatography coupled to tandem mass spectrometry (LC-MS-MS) and microarray expression profiling. Mined tissue was associated with increased concentrations of cytokinin, jasmonic acid, and salicylic acid, and a decrease of abscisic acid. Interestingly, host genes involved in cytokinin activation were significantly down-regulated whilst genes involved in cytokinin interconversion and degradation were significantly up-regulated, suggesting that cytokinin levels should have decreased. This result suggested that *Phyllonorycter blancardella* was the source of the cytokinin.

One of the most interesting recent advances in the role of phytohormones in galling was provided by Yamaguchi et al. [59]. The authors investigated the interaction between the gall-inducing sawfly (*Pontania* spp.) and its host plant *Salix japonica.* Using a combination of LC-MS-MS to quantify hormone levels, feeding experiments, salivary glands, and RT-qPCR to investigate signalling pathways within the host, the researchers demonstrated, for the first time, the ability of a galling insect to synthesise indole acetic acid (IAA) from tryptophan. The presence of high concentrations of *t*-zeatin riboside (the active precursor of *t*-zeatin) in the salivary glands is strong evidence that the insect is also able to synthesise cytokinin. 

Despite there being extensive and compelling evidence associating phytohormones, particularly auxins and cytokinins, with gall initiation and development, there is a lack of information regarding the molecular mechanisms involved. Significant cross-talk is known to occur between different hormone signalling pathways which allows for modulation of plant developmental and defence responses. It will be interesting for future studies to investigate how induced changes in hormone concentrations lead to gall formation, especially in conjunction with insect-secreted effectors. A deeper understanding of the pathways modified by these signalling components should provide a relatively comprehensive, mechanistic understanding of the interaction.

## 4. Plant Defence Responses against Galling Insects

In order to fully comprehend how an insect is able to induce a gall on its host, it is also important to understand how the plant responds during infestation. A great deal of omics data has been generated by researchers investigating plant development as well as responses to biotic and abiotic stresses. One of the primary goals of current researchers is to generate a comprehensive model of the plant system through integration of data [7,60]. One recent example of this approach is provided by Dong et al. [56] who integrated heterogeneous omics data to investigate the crosstalk between different branches of the *A. thaliana* immune system. As our understanding of the core regulatory network of plant defence improves, it will enable researchers to better understand how the host and pest interact with each other, as well as how these signals are processed within each organism.

Once a pest is recognised by its host, the information is transmitted and interpreted by signalling machinery and a coherent and coordinated response is launched. Plant defences form a robust system, which is an important property that would allow the plant to overcome pathogen or pest manipulation and elicit downstream defences [56]. These defences may be broadly divided into direct and indirect responses [61]. Induced direct defences involves the synthesis of toxic proteins or secondary metabolites that directly affect the pest whilst indirect defences involves the release of volatile components that provide a chemical cue to predators and parasitoids of the pest [61,62]. Both pathways have been shown to be important elements in plant-galling insect interactions.

### 4.1. Direct Defences

The production of defence-associated proteins is an important inducible response in plant resistance against insect herbivores. These proteins promote resistance through anti-feedant activity, either reducing nutrient quality or blocking nutrient uptake, in the insect digestive system [61,63,64]. For example, Hessian fly larvae feeding on resistant wheat presented extensive disruption of midgut microvilli which resulted in larval death [65]. A number of different protein classes have been reported to be associated with defence. Protease inhibitors are one example; these proteins act by blocking digestive enzymes in the insect gut, thereby reducing the ability of the insect to digest plant material [66]. Up-regulation of protease inhibitor genes in response to galling has been recorded in a number of interactions [23,26,28,67]. 

Plants are able to produce a wide range of chemicals to defend themselves against insect herbivory. Secondary metabolites that show insecticidal activity generally target specific insect biological systems such as the nervous and digestive systems [61]. This is supported by a transcriptome study by Zhang et al. [68] of the Hessian fly gut that showed the expression of putative detoxification genes including cytochrome P450s, glutathione *S*-transferases, peroxidases, ferritins, catalases, and peroxyredoxins in response to toxic chemicals produced by the plant upon infestation. These chemicals also participate in other defence mechanisms such as barrier reinforcement that reduce the herbivore’s ability to penetrate the plant, which is predominantly achieved through lignification of the cell walls, as well as deposition of other compounds such as suberin and callose [61]. This defence mechanism has been recorded as a response to Hessian fly larval feeding on wheat [28]. Some studies have also described the ability of galling insects to suppress the expression of genes encoding proteins that contribute to lignification, thus highlighting the importance of this response [26]. Furthermore, some galling species are able to influence the accumulation and localization of plant secondary metabolites which may allow the galler to defend itself against predators or parasitoids [69].

### 4.2. Indirect Defences

Indirect defences involve the release of volatile cues that allow the plant to interact with the pest, neighbouring plants, or predators and parasitoids of the pest [62]. Following pest recognition, the inducible indirect defence may act by attracting parasitoids of the herbivores [70] or alert neighbouring plants to the presence of the herbivores [71]. The involvement of this mechanism in plant-galler interactions has been demonstrated in a few systems. For example, Damasceno et al. [72] showed that feeding by unidentified galling psyllids caused a change in the volatile profile of *Schinus polygamous* (peppertree) and *Baccharis spicata*. Similarly, Oates et al. [23] showed that the blue gum chalcid wasp, *Leptocybe invasa*, induced changes in the volatile terpene profile of a resistant or susceptible host. Tellingly, galling insects have been shown to actively suppress the indirect responses of their hosts in susceptible interactions [4,73]; however, gall-inducers suffer high mortality rates from parasitoids, and additional studies are needed to determine the role of induced volatile changes [1].

To date, there are relatively few studies that have examined plant responses to infestation by galling insects. These studies have provided information on putative defence mechanisms that are employed by the plant during resistance and, as discussed earlier, possible pathways that are targeted by the insect during susceptibility. However, there is extremely limited information regarding the direct interactions between the pest and its host. The availability of more datasets as well as more detailed experiments, for example a time series analysis of the earliest interaction between the organisms, will allow for the development of a model plant-galling insect interaction network. This will significantly improve our understanding of the plant-galling insect interaction by facilitating comparative analyses of galling systems where limited data is available.

## 5. Host-Pest Interactions: Enter Systems Biology

**Figure 1:**
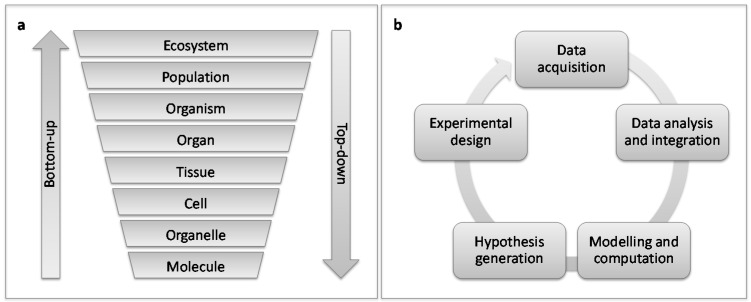
Concepts in systems biology approaches. (**a**) Holistic (top-down) vs. reductionist (bottom-up) approaches to study and integrate the various levels of biological systems; and (**b**) an iterative process that is used to generate data and model a biological system.

The interaction between a plant and a pest involves complex networks of molecular and physiological processes within and between each organism (interactome). These processes are multi-layered, stretching from molecular to organismal (Figure 1a) that all contribute to the outcome of the interaction, i.e., whether the plant launches a successful defence response, or whether the pest launches a successful infestation. Natural systems introduce a multitude of additional complicating factors to the relationship ranging from the pest’s level of specialisation and feeding guild to the ecological community and abiotic factors. Understanding plant-pest interactions at a systems level is indispensable to elucidating the mechanisms behind the interactions. However, a combined understanding of how systems components interact to determine the outcome is required for a holistic view of such a complex, multidimensional system and is becoming increasingly important for developing strategies to control existing and emerging pests [6,74].

Systems biology is the study of complex biological systems. The field is defined by an iterative cycle of multi-layered omics data generation and modelling of data that generates new hypotheses that are tested in the laboratory and used to refine the model (Figure 1b) [60,75]. Biological systems models are frequently displayed as networks, which is a useful way of understanding how the system is organised [76]. Systems biology approaches can be categorised as bottom-up and top-down, where the bottom describes the molecular interactions of molecules and the top is the holistic view of the system which is generally created by genome-wide analysis of omics data [75]. Integrating information from various datasets and types, for example gene expression and protein-protein interaction data, is a powerful method of capturing the dynamics, complexity, and emergent properties of a system [56,60].

Plant-insect interactions are increasingly being investigated using omics technologies [6]. One of the benefits of omics data is that it facilitates analysis of non-model organisms, even in cases where little or no prior knowledge of the system exists [7]. A systems level understanding of the interactions between plants and herbivorous arthropods is currently at a relatively early stage [6], perhaps few more so than the interaction between gall-inducers and their hosts. Employing a top-down systems approach to understand these interactions is key to identifying important components in the interaction and, by extension, identifying biotechnological targets aimed at improving plant resistance against insect pests.

## 6. Questions to Be Addressed Using Systems Biology Approaches for Plant-Galler Interactions

**Figure 2:**
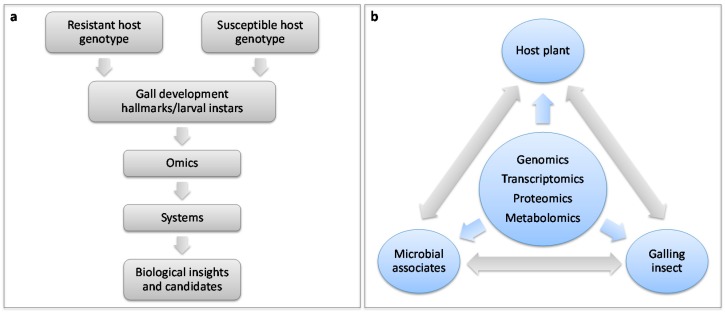
Simplified setup of a model plant-galling insect interaction and integration of multi-omics data to interrogate the interactome within and between plant-insect-microbial players during gall formation. (**a**) The development of a model system enables predictions of important candidates that are subsequently validated and used to improve the model. This resource subsequently facilitates non-model research by enabling comparative analyses; and (**b**) the integration of multiple omics datasets provides a means of interrogating the interactome within and between plant–insect–microbial players during gall formation.

In the last decade omics technologies have provided a new and potent means for improving the knowledge of plant-galling insect interactions. In order to initiate gall induction, it is essential that the insect assume control of its host’s cellular machinery. There is a growing body of evidence supporting the involvement of a diverse array of effector proteins and induced phytohormonal imbalances in galling. However, to interrogate an interaction, a controlled and reliable plant-pest system must be developed (Figure 2a). Microscopy will be a useful tool in identifying important time points during gall or insect development that can be queried using omics approaches (Figure 2a). The development of such a model system will fill an important gap in plant-galling insect research where there is currently a paucity of information linking different biological levels into a coherent system (Figure 1a and Figure 2a). The studies discussed herein typically comprised pairwise interactions between host and pest, whilst this is rarely the case under natural conditions. Investigating the targets of effectors and phytohormones, where and how they act during infestation, as well as the contribution of microbial associates to gall induction will be important questions for future studies exploring plant-galling insect interactions (Figure 2b).

A key focus for future studies is a better understanding of putative effectors that provide a means for such a wide array of insects to achieve the level of manipulation required to produce a gall. A large proportion of the Hessian fly genome is thought to encode effectors; it will be interesting to compare whether other galler genomes also encode such large effector repertoires. Similarly, it is important to understand the overlap in the targets of insect effectors. A landmark study by Mukhtar et al. [55] used proteomic data from *A. thaliana* and two pathogens to demonstrate that pathogen effectors tend to target hubs in the plant immune response network. A similar approach (Figure 2a,b) can be used to investigate whether insect effectors act similarly. Networks generated from omics data will allow for predictions of effector targets and hubs in the plant-galler interaction, as well as comparisons between different systems. Although these networks may act as guidelines in comparative research, each predicted interaction must be validated independently using functional approaches in every system (Figure 2a). Plant defences are dynamic and robust; therefore, producing a large effector suit would allow gallers to target a range of host biological processes aimed at suppressing defence and redirecting certain developmental pathways towards gall production. Understanding where and how these effectors act will help researchers develop robust, resistant plants through genetic modification or synthetic biology approaches.

While studies have demonstrated differential expression of hormone synthesis and responsive genes, as well as hormone concentrations, the regulatory functions of the hormones in many plant-galling insect interactions remain unknown [15,16,31]. Multi-omics data integration will play an important part in identifying the molecular mechanisms involved (Figure 2b). Functional studies that make use of knockout lines or hormone signalling mutants will provide a means for validating the role of the various components in the system. The ability of galling insects to independently produce phytohormones is also a long-standing hypothesis [15,16,31]. The observation that the gall-inducing sawfly (*Pontania* spp.) could synthesise IAA and cytokinin precursors was a significant breakthrough in this field [56]. Whether this is a widespread trait amongst galling insects is currently unknown, but will be an interesting comparison in the future.

Studies exploring the role of microbial associates in insect galler lifecycles are rare. Symbiotic relationships between gall-inducing insects and microbes have been hypothesised to be involved in gall development, host plant use, and the evolution of the galling trait [15,31]. The microbes undoubtedly influence plant-galling insect interactions; however, the mechanisms involved in this three-way relationship are essentially unknown. Furthermore, plant-associated microbes will also influence the system. For instance, an unknown pathogen of *Castanea* species was recently reported to cause extensive mortality in Asian chestnut gall wasp (*Dryocosmus kuriphilus*) populations [77]. Metatranscriptomics has been successfully employed to investigate bacterial associates of honey bees [78] and termites [79], as well as mutualistic behaviour between figs and fig wasps [80]. Spatial transcriptomics [81] may also have important applications in future studies in order to obtain a more comprehensive model of the interaction.

An important future aim of investigating the plant defence system is to generate a model that accurately simulates the infection or infestation which will allow for the identification of important components [7]. Current information and datasets do not yet allow this and are predominantly snapshots of single time points. Using dual RNA-Seq or metatranscriptomics in a high-resolution time course during gall development in both resistant and susceptible plant genotypes would be a powerful method to generate gene regulatory networks for host, pests, and associates. Time series analyses allow for the examination of the sequence of events during an infestation through gene expression dynamics which enables modelling of causal interactions and prediction of master regulators [7,82,83]. Furthermore, this approach is readily applicable to non-model systems and will be an invaluable step towards elucidating the interactome in the plant-galling insect interaction (Figure 2). Once a model system has been generated, studies that are limited to a single or a few datasets can make use of comparative analyses to gain biological insight.

The integrative nature of systems biology approaches provides more power in prediction of key targets for functional testing. Knowledge of key components in the plant-insect galler system and their behaviour during the interaction will help identify the most beneficial targets for improved pest management programs. For this purpose, the prediction of important candidate genes that can be explored in more targeted functional genetics experiments will be important. Pending functional characterisation, such targets could be screened for in breeding populations to identify potentially resistant material or adopted as targets of genetic modification using genome editing or RNAi approaches [84,85].

## Abbreviations

R	Resistance
PTI	Pattern-triggered immunity
MAMPs	Microbe-associated molecular patterns
ETS	Effector-triggered susceptibility
ETI	Effector-triggered immunity
HAMPs	Herbivore-associated molecular patterns
EAMPs	Egg-associated molecular patterns
*SSGP*	Secreted salivary gland protein-encoding gene
miRNA	microRNA
IAA	Indole acetic acid
LC-MS-MS	Liquid chromatography coupled to tandem mass spectrometry
